# 

**Published:** 1859-10

**Authors:** 


### EDITORIAL DEPARTMENT.

*American Convention of Dentists.*—We publish in another part of the present No., a report of the Proceedings of the last meeting of the American Dental Convention, held at Niagara Falls, on the 2d, 3d, 4th and 5th of August. As we were unable to attend the Convention, Dr. C. W. Harvey, of Buffalo, was kind enough to secure the services of a gentleman of that city to report the proceedings for the Journal. For this courtesy we are under obligations to Dr. H., and beg to return him our sincere thanks.

The second paper read by Prof. Taylor, we have not received, nor the paper by Dr. Wilson, of Boston. We shall be compelled therefore, to defer their publication until our next issue.

*American Dental Association.*—This is the designation of a new society of dentists, organized on the evening of the 3d of August. All that we know of the new association is obtained from the following notice made by our reporter for a daily paper. The next meeting is to be held in Washington City, D. C., the last Tuesday in July, 1860. When we see the constitution and learn the objects of the association, we may have occasion to speak of it again. [Eds.

During the evening of Wednesday, August 3d, a preliminary meeting was held at the International Hotel, for the purpose of organizing a new body of representative dentists. Dr. Allport, of Chicago, was appointed chairman. A committee of three was appointed to draft a constitution. This committee consisted of Professor J. H. McQuillan, of Philadelphia, Dr. Richardson, of Cincinnati, and Dr. Wright of Pittsburg.

The committee, through their chairman, Dr. McQuillan, reported Thursday a constitution, which was accepted and referred back to the committee for any emendations that might suggest themselves, until November next, when it will be published in the dental journals. Five persons were appointed by the Chair to read essays on different subjects at the first meeting of this, to be called "American Dental Association," to be held at Washington, D. C., on the last Tuesday in July, 1860. These are Drs. Taft, Watt, Richardson, of Cincinnati, Professor McQuillen, and Dr. Buckingham, of Philadelphia. These gentlemen have already indicated the subject of their papers.

*Selected Articles.*—Several selected articles, designed for the present No. of the Journal, though in type, are excludad for want of room—all the pages being filled with original communications, and the report of the proceedings of the late Dental Convention.

*To Subscribers.*—The editors respectfully request that all communications containing money, or in any way relating to the business affairs of the Journal, be sent direct to the publishers, Messrs. Lindsay & Blakiston, 25 South Sixth street, Philadelphia. The books of the Journal are in their possession, and all business letters received by the editors are forwarded immediately to them. See standing notice to readers and correspondents, in front of each No. of the Journal.

### INDEX.—VOL. IX.

PAGE.

A.

Anatomical Material, Preserving and Preparing, by W. G. Smull, M. D., . . . . 29
Application of Galvanism, Surgical, by Brown Sequard, . . . 40
Ancients, Dentistry among, . . 57
Artificial Dentures, by Dr. J Allen, 115
Acid, Nitric, and Caries, . . 141
American Dental Convention, 148, 592
Anomalous Affections of the Teeth by J. Smith Dodge, Jr., M. D., D. D.S., . . . . 207
Address on Metallurgy, by Dr. Leslie, . . . . 238
Abdomen, Iron Rod pushed through, 279
Alkaline, Dentifrice, . . . 289
Anatomy, Descriptive and Surgical, 290
Abstract of Medical Sciences, Half Yearly, . . . . 293
Antiquities, Dental, . . 294, 354
Association, Michigan State Dent., 297
Anæsthesia by Compression, 299
Alveolar Abscess, by H. N. Wadsworth, D. D. S., . . . 329
Adulterations of Food, by J. Scoffern, M. D., . . . 425
Army and Navy Dentists, importance of, . . . . 444
Academy of Medicine, . . 447
Allen, Dr. J., on Artificial Dentures, 115
Abscess, Chronic, . . . 491
American Dental Convention, Proceedings of Fifth Annual Meeting of, . . . . . 560
Application of Arsenious Acid to Teeth, . . . . 593

B.

Baltimore College of Dental Surgery, Commencement of, . 288
Buisch, W., on Digestion, . . 399

PAGE.

Bernard, M. Cl., Nerves which produce Variations in Color of Venous Blood, . . . 280
Bunon, Robert, Notice of Life of, 234
Bourdet, Notice of Life of, . 234

C.

Cancer of Inferior Maxillary, 36
Cancerous Tumor in Parotid Region, 38
Cheoplastic Method of Mounting Teeth, . . . . 57
College of Dentists, Monthly Meeting of, . . . . . 63
Carpenter on Principles and Practice of Dental Surgery, . 109
Convention, American Dental, 148
Correspondents, to, . . 149
Clark, John S., D. D. S., Paper read before Academy of Science, 178, 504
Clasps for Teeth, New Method of forming, . . . . 210
Case of Necrosis of Upper and Lower Jaw, . . . . 227
Crosby, D., M. D., Operation for Removal of Superior Maxillary, 269
Convention, Indiana State Dental, 297
Contributors, Our, . . . 300
Correspondence, European, 347, 540
Chloroform and Ether, . . 396
Carnochan, Prof. J. M., M. D., Exsection of Trunk of Second Branch of Fifth Nerve, . . . 253
Cheoplasty, . . . . 525

D.

Dental Associations, brief Review of, . . . . . 16
Disarticulation of Lower Jaw, . 34
Dentistry Among the Ancients, 43
Dental Society, Meeting of Western, 58
Development and Structure of Human Teeth, . . . 594

Page.
Dental Cosmos, . . 597
Dentures, Artificial, . 115
Dental Register, . . 147
Dental Convention, American, 148
Development of Human Teeth, by M. Magitot, M. D., 153, 301, 449
Dodge, J. Smith, Jr., M. D., D. D. S., Anomalous Affections of the Teeth, . . . . 207
Duval, M., Notice of Life of, 233
Dental College, Commencement of Baltimore, . . . . 288
Dentifrice, Alkaline, . 289
Descriptive and Surgical Anatomy, 290
Dental Antiquities, . 294, 354
Dental Association, Michigan State, 297
Dental Convention, Indiana State, 297
Dental Science, London Quarterly Journal of, . . 299
Diseases of Mouth in Infancy, by W. G. Smull, M. D., . 226
Dental Surgery, Review of, 384
Dentists, Army and Navy, . 444
Deleterious Effects of Swilled Milk, 447
Dental Neuralgia, . . 448
Dental Surgery, . . . 448
Dental Surgery, System of, by John Tomes, F. R. S., Review of, 546

E.

Electricity in the Extraction of Teeth By D. P. Gregg, D. D. S., 236
Exsection of Trunk of second Branch of Fifth Nerve, . . 253
European Correspondence, 347, 540
Extracting Teeth by Electricity, Philosophy of, . . 212
Essay, by Prof. James Taylor, M. D., . . . 526
Errata, . . . 599

F.

Filling Teeth. By Samuel Mallet, D. D. S., . . . . 188
Forbes, Dr. Isaiah, Letter from, 250
Faudiguire, Leroy de la, Sketch of Life of, . . . 235

G.

Gold Foil, . . . . 597
Galvanism, Surgical Application of 40
Gold Plates, Ingalls on Warping of, 250
Green Wall Paper, Poisoning by, 289
Gorgas, F. G. S., D. D. S., Ideas on Mechanical Dentistry, . 333
Georgia Dental Society, . 593

Page.
H.

Heyfelder, M., on Disarticulation of Lower Jaw, . . . 34
Hill, A., D. D. S., Odds and Ends, by, . . . . 198
Hist., Review of Dental Surgery, 384
Humby, J. M., on Improved Model Tray, . . . . 394

I.

Inferior Maxillary, Resection of, 36
Items, Professional, . . 150
Ingalls on Warping of Gold Plates, 250
Iron Rod Pushed through Abdomen, . . . . 279
Ideas on Mechanical Dentistry, 333
Irregularity of Teeth, . . 343

J.

Journal of Dental Science, London Quarterly, . . . 299
Johnston, Christopher, M. D., on Odontology, . . . 337

K.

King, John, M. D., Microscopist's Companion, . . . 442

L.

Letter from M. Heyfelder, on Disarticulation of Lower Jaw, 34
Lecture on Principles and Practice of Dental Surgery, . . 109
Lateral Pressure of Teeth, in relation to Obscure Nervous Affections, . . . 124
Le Maire, Sketch of Life of 232
Laforgue, Lewis, Sketch of Life of, 235
Letter from Dr. Forbes, . 251
London Quarterly Journal of Dental Science, . . . 279
Loss of Teeth, . . 448

M.

Maxillary Sinus, Mucous Polypi of, . . . . . 32
Meeting of Western Dental Society, 58
Monthly Meeting of College of Dentists, . . . . 63
Mallett, D. D. S., on Filling Teeth, 188
Malformation, Remarkable, . 244

Page.
Metallurgy, Address on, . 238
Michigan State Dental Association, 297
Magitot, Emile, M. D., on Human Teeth, . . 153, 301, 449
Mechanical Dentistry, . 333
Model Tray, . . . 394
Microscopist's Companion, 442
M'Calla, J., D. D. S., on Lateral Pressure of Teeth, . 124

N.

Nitrogen or Azote, . . 3

Nursing after Operation for Hare Lip, . . . . 278

Note upon Odontology, . 337

Narcotism, Voltaic, . . 445

O.

Odds and Ends, . 198, 501
Operation for Removal of Upper Jaw, . . . . 269
Observations on Use of Chloroform and Ether, . . . 396

P.

Parmly, L. S., Obituary of . 598
Preserving and Preparing Anatomical Material, . . . 29
Philosophy of Extracting Teeth by Electricity, . . . 212
Periosteum, Function of, . 446
Public Benefactor, Trials of, . 292
Prizes, . . . 593

Q.

Quarterly Journal of Dental Science, . . . . 299

Page.

R.

Researches on Restoring Life to Individuals dying of Disease, 434

S.

Surgical Application of Galvanism, 40
Successful Removal of Upper Jaw, 269
Surgery, Dental, . . 448
Sudderth, Dr. S. A., Obituary, 300
Smith, J. B., M. D., Letter from, 300
Smull, W. G., M. D., on Diseases of the Mouth, . . 326

T.

Teeth, Development of Human, 153, 301, 449
Teeth, Filling, . . . 188
Teeth, Anomalous Affections of, 207
Teeth, Clasps for, . . 210
Teeth, Extracting, by Electricity, 212, 236
Teeth, Retention of, after Necrosis of Jaw, . . . 290
Teeth, Loss of, . . 448
Townsend, Dr. Elisha, Obituary, 151
Taylor, Prof. James, M. D., Essay read by, . . - 526
Tomes, John, F. R. S., System of Dental Surgery, . . 546
Tomes' Dental Surgery, . 595

U.

Upper and Lower Jaw, Necrosis of, . . . . 227

